# Polymer-Assisted Graphite Exfoliation: Advancing Nanostructure Preparation and Multifunctional Composites

**DOI:** 10.3390/polym16162273

**Published:** 2024-08-10

**Authors:** Jaime Orellana, Esteban Araya-Hermosilla, Andrea Pucci, Rodrigo Araya-Hermosilla

**Affiliations:** 1 Programa de Doctorado en Ciencias de Materiales e Ingeniería de Procesos, Universidad Tecnológica Metropolitana, Ignacio Valdivieso 2409, San Joaquín, Santiago 8940577, Chile; jaime.orellanao@utem.cl; 2 Departamento de Ingeniería Química, Biotecnología y Materiales, Facultad de Ciencias Físicas y Matemáticas, Universidad de Chile, Beauchef 851, Box, Santiago 8370456, Chile; esteban_araya84@yahoo.es; 3 Dipartimento di Chimica e Chimica Industriale, Università di Pisa, Via Moruzzi 13, 56124 Pisa, Italy; andrea.pucci@unipi.it; 4 Instituto Universitario de Investigación y Desarrollo Tecnológico (IDT), Universidad Tecnológica Metropolitana, Ignacio Valdivieso 2409, San Joaquín, Santiago 8370456, Chile

**Keywords:** graphite, functional polymers, exfoliation, graphite nanoplatelets, few layers graphene, nanocomposite, multi-functionality

## Abstract

Exfoliated graphite (ExG) embedded in a polymeric matrix represents an accessible, cost-effective, and sustainable method for generating nanosized graphite-based polymer composites with multifunctional properties. This review article analyzes diverse methods currently used to exfoliate graphite into graphite nanoplatelets, few-layer graphene, and polymer-assisted graphene. It also explores engineered methods for small-scale pilot production of polymer nanocomposites. It highlights the chemistry involved during the graphite intercalation and exfoliation process, particularly emphasizing the interfacial interactions related to steric repulsion forces, van der Waals forces, hydrogen bonds, π-π stacking, and covalent bonds. These interactions promote the dispersion and stabilization of the graphite derivative structures in polymeric matrices. Finally, it compares the enhanced properties of nanocomposites, such as increased thermal and electrical conductivity and electromagnetic interference (EMI) shielding applications, with those of neat polymer materials.

## 1. Introduction

Graphitic nanofillers such as graphene have significantly advanced composites nanotechnology [1,2,3]. Graphene is a two-dimensional crystal of carbon atoms united by sp^2^ sigma bonds. The sp^2^ orbitals that do not contribute to the crystal frame create the π-bonds network responsible for its outstanding electrical properties [4]. The honeycomb-like structure of graphene presents a two-dimensional lattice with extremely efficient characteristics, including a superficial area of 2630 m^2^/g [5], a Young’s modulus of a single layer close to 2 TPa [6], and electric and thermal conductivities of 1.4 × 10^−6^ S/cm [7] and 5000 W/m-K [8], respectively. With its high specific surface area, thermal conductivity [9], mechanical strength [10], negative thermal expansion [11], and optical transparency [12], graphene has found applications in a wide array of fields, including supercapacitors [13], antibacterial scaffolds [14], photovoltaic cells [15], biosensing [16], and transparent electronics [17].

Remarkably, the behavior of graphene is significantly influenced by the source material from which it is synthesized, its resulting nanoscale structure and dimensions, and its interfacial interactions with the substrate on which it is stabilized. These factors are determined by the manufacturing method employed to produce it [18]. From the available literature, two main approaches for graphene formation can be distinguished: (1) The bottom-up approach to graphene synthesis involves assembling small carbon units onto a substrate. The most common techniques include chemical vapor deposition (CVD), epitaxial growth, and pyrolysis. In CVD, gaseous reagents react on the surface of a metal substrate at high temperatures, resulting in the deposition and assembly of graphene. This method offers several advantages, including high structural quality, large surface area, good reproducibility, and precise control over the number of graphene layers formed [19]. Pyrolysis, similar to CVD, involves the decomposition of low molecular weight carbon-containing compounds in the absence of oxygen, typically using a noble gas plasma. The resulting carbon is then deposited onto a glass substrate. [20]. Epitaxial growth involves the thermal decomposition of silicon carbide (SiC) in a vacuum or argon atmosphere, where silicon sublimes, leaving carbon atoms to form high-quality graphene sheets on the surface of the SiC [21]. Although these methods produce high-quality graphene, they have limitations such as the limited availability of materials, high energy consumption, and significant challenges for large-scale industrial application due to associated costs [22]. Conversely, (2) the top-down approach involves the use of bulk graphite that is then subjected to dynamic forces to reduce its size until nanostructures are obtained. Techniques for this include physical, chemical, and electrochemical exfoliation [23,24].

The literature clearly demonstrates the excellent properties that graphene can impart to polymer composite materials, such as enhanced electrical and thermal conductivity and increased mechanical strength, compared to the intrinsic properties of the polymers alone. [5]. However, the inherent stacking behavior of graphene in its 2D plate form makes it difficult to disperse in polymer matrices. This challenge arises primarily from the lack of strong interfacial adhesion between the polymer and graphene at the molecular and nanoscale levels. Poor interfacial adhesion leads to the formation of graphene clusters, which impede the materials´ optimal function as a filler when creating percolative pathways for energy transfer. Once strong interfacial interactions are achieved, the extraordinary properties of graphene can be effectively transferred to polymer matrices, enhancing their performance and functionality.

Despite graphene’s many beneficial properties, its current market price remains exceptionally high, making it less economically viable than cheaper graphitic fillers such as graphite or carbon black in polymer composites. Consequently, fundamental research and R&D departments have developed various methods to produce graphene for different technological and commercial purposes [18].

In situ graphene production from graphite has been proposed to formulate feasible low-cost graphene-based polymer composites. According to previous research, polymers can facilitate the intercalation and exfoliation of micron-sized graphite into modified nanostructures such as graphite nanoplatelets, few-layer graphene, or graphene through various physical and chemical approaches. For instance, researchers have reported the use of a solvent blend mix [25], melt mixing in conventional thermal reactors [26], shear force in twin-screw mixer [27], and normal force in ball milling [28]. The main challenge of these techniques is to detach graphite sheets from each other (e.g., by overcoming van der Waals forces), continuously transforming the micrometric graphite structure into modified nanostructures. In addition, the selected polymer matrix must ensure good dispersion and stabilization of the exfoliated graphite layers through physical and/or chemical interactions within the polymer matrix. The intercalation/exfoliation of graphite by polymers involves inserting polymer chains between graphite layers, weakening van der Waals forces, and facilitating the separation into graphene sheets. The choice of polymer and its functional groups is crucial for ensuring effective interactions with graphite at the atomic and nanoscale interface. Common polymers include polypropylene [29], polyvinyl alcohol (PVA) [30], polystyrene (PS) [31], and polyvinylpyrrolidone (PVP) [32]. Methods of polymer intercalation include solution blend mixing [32], melt processing mixing [33], and ball milling [34]. The properties of the resulting composite are influenced by the loading ratio and type of interfacial interactions, which can be physical (hydrogen bonds, van der Waals forces) or chemical (covalent bonds) [35,36]. Extrusion and the discontinuous Brabender-type mixing are used to process polymer/graphite composites, providing a high shear force for effective exfoliation and homogeneous dispersion of graphene derivatives [37,38,39,40,41,42,43]. These methods allow for large-scale production under mild conditions and also open possibilities for in situ covalent bondsbetween graphene and polymer chains [29].

This review examines different methods for the top-down exfoliation of graphite into smaller graphitic structures such as graphite nanoplatelets, few-layer graphene, and graphene-assisted polymers. While there are several excellent reviews covering the intercalation polymerization of monomers to produce expanded graphite during the in situ production of polymer nanocomposites [44,45,46,47,48,49], methods describing the chemistry involved during graphite’s exfoliation process, emphasizing the interfacial interaction, dispersion, and stabilization of the exfoliated modified forms of graphite in post-polymerized thermoplastics and thermoset resins, have not yet been reviewed. Particularly, it has been found that steric repulsion forces, van der Waals forces, hydrogen bonds, π-π stacking, and covalent bonds, in conjunction with shear forces, play crucial roles in the exfoliation, dispersion, and stabilization of exfoliated graphene derivative structures. These interactions prevent aggregation and facilitate the generation of new composite materials.

**Scheme 1:**
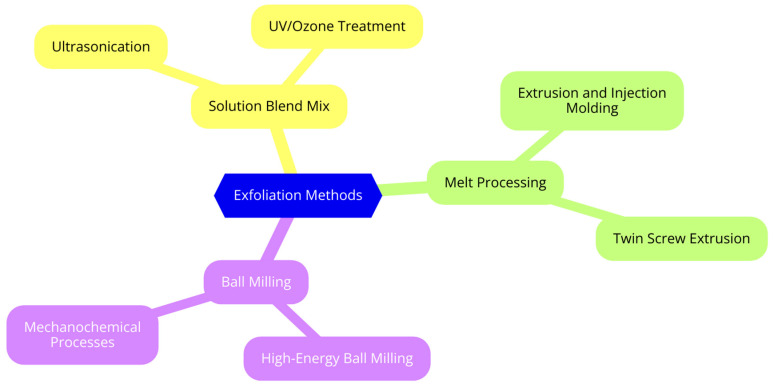
Engineering state-of-the-art techniques to generate exfoliated graphite/polymers composites.

Additionally, this review covers advanced engineering techniques, including solvent blend mixing, melt mixing in conventional reactors, twin-screw mixing, Brabender mixing, and ball milling (see Scheme 1). It also discusses the enhanced and unique properties of these materials, such as an increased modulus in structural nanocomposites and improved thermal and electrical conductivity for applications in electromagnetic interference (EMI) shielding.

## 2. Top-Down Exfoliation of Graphite

**Figure 1:**
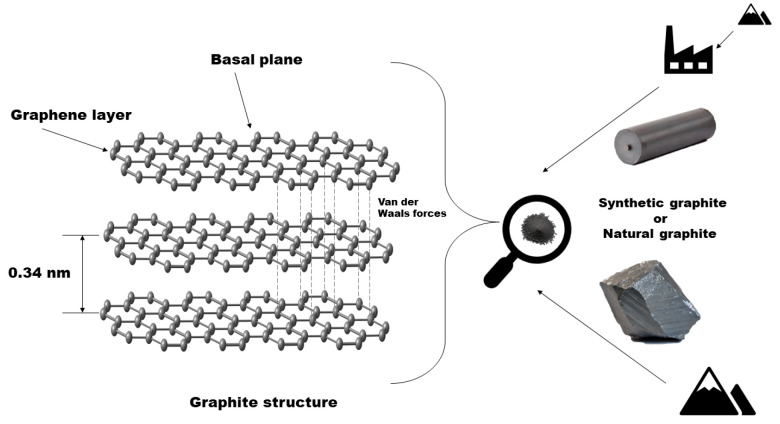
Graphite structures and sources.

The top-down exfoliation of graphite is a method for obtaining graphene sheets from 3D crystalline graphite [24]. Graphite, consisting of carbon layers (graphene layers), is primarily sourced from mining, with natural graphite being extracted from the earth. China, India, and Brazil are the leading producers. Another method of obtaining graphite is through the production of synthetic graphite, which is produced via a process called graphitization. In this process, carbon-rich materials are subjected to high pressures and temperatures. Both synthetic and natural graphite share the same laminar structure, which is held together by van der Waals interactions generated by the delocalized π-orbitals, giving them similar properties for industrial applications (Figure 1) [50,51]. Graphite is an anisotropic material with excellent thermal and electrical conductivity within the layers but poor conductivity across the layers. For instance, the thermal conductivity of graphite is high in the layer plane with a value of 2000 Wm^−1^K^−1^, but only 7 Wm^−1^K^−1^ across the layers [52]. Moreover, the thermal conductivity of graphite depends on the thickness of the graphene stacks. Thinner graphite structures show higher thermal conductivity, which decreases as the thickness increases [53]. Graphite has numerous applications in electronics, optoelectronics, electrocatalysis, batteries, supercapacitors, solar cells, and photocatalysis [54].

In a general context, the top-down techniques currently used to separate graphite into graphene or graphite micro/nanostructures via exfoliation mainly rely on mechanical force or chemical/electrochemical routes. Compared to bottom-up approaches, the scalability of the top-down processes is viable, but the quality of the graphitic structures obtained is lower [55].

The methods used to generate graphene from graphite via exfoliation (top-down methods) are broadly classified into two categories: liquid-phase-exfoliation (LPE) and dry-exfoliation (DE) [56]. LPE involves using chemical routes and/or cavitation forces to promote the delamination of graphite and the separation of sheets to produce graphene [57].

Exfoliation via the oxidation and reduction routes starts by oxidizing graphite with concentrated acids to obtain graphite oxide and then, after an easy exfoliation, graphene oxide (GO). Subsequently, the use of reducing agents helps the producer obtain reduced graphene oxide (rGO), a process required to restore most of the typical features of graphene [58]. The resulting exfoliated graphene layers can be easily dispersed in water or polar organic solvents. One commonly used reducing agent is phenylhydrazine, which effectively removes the oxygen groups of GO [59]. However, it is important to note that hydrazine is expensive, toxic, and has a lengthy synthesis route [55]. Furthermore, rapid reduction kinetics reduce only a small portion of the GO, leading to the formation of low-quality graphene sheets [55,59,60]. However, there are numerous other methods to chemically reduce GO, including borohydrides, aluminum hydride, and amino acid derivatives, to name a few [61].

Electrochemical exfoliation involves using a pristine graphite anode and a noble metal cathode (such as Pt), or vice versa, to apply a voltage that drives the movement of the electrolyte (intercalating species) into the interlayer of graphite within a catalytic cell. The intercalation of ionic species weakens the van der Waals forces between the layers, facilitating their separation [60]. The main advantage of this method is the relatively clean physicochemical process. Also, with the help of sonication, electrochemical exfoliation can optimize the introduction of ions into the graphite lattice, thus producing single or few-layer graphene [62]. The exfoliation process via electrochemistry can also effectively generate functionalized graphene [63]. Zhang and co-workers reported a straightforward approach to exfoliating graphite by expansion. This method involves intercalating a chemical compound (LiOH) between the graphite sheets. Then, the material is exposed to high temperatures, causing the intercalated compound to enter the vapor phase and expand the graphite, leading to exfoliation [64]. Zhu and co-workers presented a liquid-phase exfoliation of graphite using organic solvents, H_2_O/surfactant, or ionic liquids assisted by sonication. It is an attractive technique due to its low cost, but displays disadvantages such as high polydispersity, low yields, and lingering solvent traces that are difficult to remove [65].

The DE uses physiochemical or mechanical force to exfoliate graphite into graphene layers. As previously mentioned, crystalline graphite is an ideal starting material due to its composition, which is based on carbon atoms covalently bonded and forming a honeycomb lattice structure. In graphite, attractive interactions such as van der Waals forces unite the lattices within the micron-sized structure. Therefore, the attractive forces must be overcome to exfoliate the stacked sheets of graphite successfully. In graphite, the graphene sheet exfoliation (one mono-atomic layer) requires a mechanical cleaving force higher than 300 nN/µm^2^ [66]. Therefore, using physiochemical or mechanical force for exfoliation enables the production of laminar structures with nanometric dimensions [67]. The exfoliation products must have a size of less than 100 nm on the Z axis, but a size larger than 100 nm is allowed on the X and Y axes. The exfoliation can generate monolayer materials, a few stacked sheets, or multi-layer stacks with dimensions of less than 100 nm [68]. The most representative techniques used to exfoliate graphite are micromechanical cleavage [69], the anodic bonding method [70], and selective laser ablation to obtain graphene layers [71]. These methods promote the division of crystalline graphite along its crystallographic planes. Pirzado et al. [72] presented an excellent example of DE via mechanical exfoliation by ablation process. The method can be perceived as a pencil lead rubbed using a glass disk with a rough surface. In this case, a pure graphite disk spinning on the glass disk underwent mechanical ablation, generating the graphitic graphene-like nanostructures. The resulting powders were dispersed in ethanol to purify the product, and the mixing was left to settle. The supernatant showed a few graphene layers with a yield of around 60%. Methods for exfoliating and stabilizing graphene with natural and bio-based materials have also been explored [73]. These approaches were mainly applied to expandable graphite via exfoliation by shear force or sonication in different molten matrices like Candelilla wax [74] and beeswax [75]. The use of such eco-friendly materials will pave the way for developing sustainable processes of graphite exfoliation.

Exfoliated graphite (ExG) has attracted considerable attention as an effective material for electromagnetic interference (EMI) shielding due to its distinctive structure and high electrical conductivity. Indeed, with the ongoing advancements in global communications technology, electromagnetic interference has emerged as a significant issue affecting electronic devices and human health [76]. Consequently, adequate protection against electromagnetic radiation has become a substantial topic for research during the last two decades [77]. The separation of carbon layers during the exfoliation process enhances connectivity and the preferred orientation of the carbon layers, enabling the reflection and absorption of electromagnetic radiation. This unique structure significantly increases the material’s EMI shielding effectiveness, making ExG suitable for applications such as EMI gasketing and lightweight shielding solutions for electronic devices [78]. Various fabrication methods, including blending, in situ polymerization, and layer-by-layer assembly, improve the composites’ properties, making them ideal for flexible electronics, sensors, and supercapacitors [79,80].

The experimental characterization of graphene is crucial to determining its fundamental properties, such as thickness, defect density, chemical composition, and the crystallographic structure obtained after graphite exfoliation. Techniques like Raman spectroscopy, X-ray photoelectron spectroscopy (XPS), and X-ray diffraction (XRD) provide essential data that inform the understanding of graphene’s electrical, thermal, and mechanical performance. This detailed characterization is vital for tailoring graphene for specific applications, including electronics, sensors, and composite materials, ensuring optimal functionality and performance in these advanced technological fields.

**Raman spectroscopy**

Raman spectroscopy is a highly effective technique for studying graphene derivatives due to its strong resonance Raman scattering, providing robust signals from even a monolayer of graphene, making it ideal for material characterization. In the Raman spectra of graphene and graphite, three main characteristic bands are observed. The D band (~1330 cm^−1^) is associated with edges and defects (sp^3^ carbons). The G band (~1580 cm^−1^) is assigned to sp^2^ carbons, and the G’ (2D) band (~2650 cm^−1^) is the second-order overtone of the D band. The shape and position of the G’ band provide detailed information about the number of graphene layers, making Raman spectroscopy valuable for determining the number of layers and their graphitic purity. Studies by Dresselhaus et al. demonstrated that the 2D band evolves, broadening and shifting upward as the number of layers increases. The intensity ratio between the D and G bands (ID/IG) is a crucial indicator of defect levels in graphene. For graphene produced by intercalation/exfoliation methods, this ratio is typically higher than in the original graphite, reflecting damage incurred during exfoliation, the formation of new edges, and changes in carbon hybridization. In pristine graphite, the D band (~1325 cm^−1^) is related to the mean crystallite size, while the G band (~1570 cm^−1^) correlates with the distance between point defects in the crystalline lattice. Upon exfoliation, a weak D band at 1341 cm^−1^ and a sharp G band at 1563 cm^−1^, along with a more symmetric 2D band at 2691 cm^−1^, indicate the production of graphene. A relatively weak D band and lack of broadening of the G band suggest a low defect density, likely associated with edges rather than structural disorders within the graphene plane. Therefore, Raman spectroscopy is an indispensable tool for evaluating and developing polymer-assisted graphite exfoliation materials, providing essential insights into the structural quality and characteristics of intercalated/exfoliated graphene [27,81,82,83,84,85].

**XPS**

X-ray Photoelectron Spectroscopy (XPS) is essential to analyses of the surface chemistry and functionalization of exfoliated graphite, providing detailed information on elemental composition and chemical states. It identifies oxygenated functional groups (e.g., C–C, C=O, OC=O, C–O) and detects non-covalent interactions such as π-π* bonding with other molecules, aiding in stabilizing and dispersing exfoliated layers. XPS reveals changes in atomic concentrations post-treatment, indicating increased surface functionalization, which enhances bonding and reduces interfacial debonding in composites, ultimately improving mechanical and electrical properties. This makes XPS crucial for optimizing exfoliation processes and developing advanced graphite-based materials [25,83,86].

**XRD and WAXD**

X-ray diffraction (XRD) is essential for evaluating the exfoliation of graphite in composites. XRD identifies crystal planes and determines the phase structures and exfoliation degrees. Key peaks, especially the (002) around 2θ ≈ 26.5º, indicate interlayer spacing and crystalline order. Increased interlayer spacing and decreased peak intensity signal effective exfoliation and disruption of the graphite structure. Additives like polyurethane or high-energy ball milling can significantly promote exfoliation, which is reflected in changes in diffraction patterns. For instance, extended milling time reduces crystallinity and increases disorder in graphite layers. XRD also reveals the conversion of stable hexagonal phases to turbostratic phases in exfoliated graphite, indicating improved exfoliation and dispersion in the composite matrix. In summary, XRD is crucial for characterizing and optimizing graphite exfoliation processes, providing valuable data on the structure and exfoliation degree of graphite sheets in various composites [28,87,88,89,90]. Wide-Angle X-ray Diffraction (WAXD) is an essential technique for evaluating the structure, expansion, and exfoliation of graphite particles in composites. By analyzing WAXD patterns, researchers can determine interlayer spacing and exfoliation levels. Changes in the (002) diffraction peak’s position or intensity indicate modifications in graphite layers, with a smaller angle and larger d-spacing suggesting partial exfoliation and reduced peak intensity indicating better exfoliation. Various intercalation methods and phase transitions significantly impact exfoliation, as evidenced by shifts in peak intensity and position. Higher processing temperatures also enhance exfoliation, leading to better dispersion of graphite layers. Overall, WAXD provides critical insights into the effectiveness of exfoliation techniques and processing conditions on graphite composites [27,29,91,92].

## 3. Exfoliated Graphite at the Polymeric Interface/Interphase

Graphite-based polymer composite materials are constantly evolving thanks to advances in polymer processing techniques and a greater understanding of advanced carbon nanofillers and the interface/interphase around them [93]. Notably, polymer/graphite composites, under development for many years, have already found diverse applications in electronic electrodes, resistors, dye-sensitized solar cells, packaging, sensors, flame-retardants, and membranes [46]. A fascinating example is using polyamide, polyimide, and poly(amide-imide) mixed with graphite to generate nanocomposites that find applications in fields like the automotive industry, aerospace, packaging, sensors, and microelectronics [94]. Other remarkable examples include using exfoliated graphite to produce composite materials for films, conductive inks [95], and modified asphalt to improve its temperature stability [96]. While the topic of polymer intercalation into graphite layers via in situ polymerization to produce graphene/polymer nanocomposite is not covered in this review, it is worth mentioning for its potential [44]. For instance, poly(methyl methacrylate) produces reinforced composites with graphene, graphene oxide, and graphite via in situ polymerization during melt blending [97].

The loading ratio and the interactions at the interfacial region between the components play a vital role in determining the properties of the composites. The interfacial region, which is the boundary layer where the polymer matrix and the filler interact, is crucial to defining the overall performance of the composite material. [98]. Graphite structures contribute to additional properties in composite materials due to their high electronic density from delocalized π bonds and their large surface area, which enables significant interfacial interactions with polymer matrices. These interactions can be physical, such as hydrogen bonds and van der Waals interactions, or chemical, involving covalent bonds. The combination of various interfacial interactions results in a broad spectrum of new properties in the composite. Interfacial covalent and physical interactions between the matrix and the graphite enhance filler dispersion and stabilization within the matrix [99,100]. Additionally, covalent and physical bonding between the matrix and the filler imparts new characteristics to the material. For covalent functionalization between the matrix and the filler, grafting-from and grafting-to techniques are commonly used to modify the interfacial properties of these materials [101].

For instance, the interfacial region of these materials has been investigated for biocompatibility due to the high number of interactions between the graphitic material and biomolecules. This establishes the field of nanobiotechnology, which remains highly attractive today for applications in brain electrodes, nervous systems, and artificial muscles. [102,103,104].

To achieve a higher surface area in a composite material, the filler must be dispersed and stabilized throughout the matrix, avoiding the formation of agglomerations that reduce the interfacial area. In this context, interfacial interactions play an essential role by keeping the filler dispersed and stable within the matrix. Specifically, for lamellar graphite structures, the stacking forces of π bonds must be overcome to exfoliate the graphitic structures. Exfoliation of graphite can be defined as the process in which the graphene layers are separated from the crystallographic planes along the c-axis of graphite. [105]. It generates puffed-up materials with a low density and high-temperature resistance. This can be used as a filler for composites. For instance, the first composite with exfoliated graphite was reported in 1915 by J. W. Aylsworth (US Patent 1 137373) [106], who combined exfoliated graphite material with a thermosetting polymer [107]. This discovery marked a significant milestone in the field. However, the study of 2D material has gained more attention and interest worldwide since 2004, when graphene was successfully obtained [108].

In a general context, optimizing composite materials production processes by using in situ exfoliation of graphite into graphene-like structures would reduce costs in energy consumption and reagents. During in situ polymer-assisted exfoliation of graphite, techniques such as sonication, shear force, or thermal mixing may affect the electrical and thermal properties of the final composite material [109]. This is mainly due to the decreasing filler size, which affects the final percolative network. If there is a covalent interfacial interaction between the matrix and filler, it may interrupt the filler’s π conjugation. Although this favors the filler/polymer compatibility, it also decreases the filler’s conductivity due to the change in carbon hybridization from sp^2^ to sp^3^ [110]. Another factor that influences the percolative networks is the orientation of the filler. The orientation is essential because the percolative response might be unstable, making spontaneous changes when the filler is randomly oriented. The latter strongly depends on the matrix material (functionality) and the processing techniques. For instance, sensing technology is one of elastomeric composites’ most significant technological applications [99]. These materials can measure deformation because of their piezo resistivity properties [47]. Achieving homogeneous electrical conduction in composite systems containing graphite fillers involves several key factors. These include the functionalization of the polymer matrix to enhance compatibility with the fillers, the formation of percolative networks to ensure continuous pathways for electrical conduction, and strong interfacial interactions between the polymer and the graphite fillers to maintain dispersion. Additionally, mechanisms such as electron hopping and tunneling are crucial for facilitating charge transfer and preventing filler agglomeration [111].

Graphite nanoplatelets (GNP) [24], i.e., nanoscale particles derived from graphite and characterized by their thin, flat, plate-like structure with high aspect ratios, play a pivotal role in the generation of polymer nanocomposites. They consist of multiple layers of graphene, each layer being an atom-thick sheet of carbon atoms arranged in a honeycomb lattice. When dispersed in polymers, they significantly enhance their mechanical properties, such as wear resistance, and introduce novel features to the composite, such as high electrical and thermal conductivity [37]. Developing new dispersion techniques is crucial to prevent the filler’s aggregation; otherwise, it behaves more like graphite filler [38]. The percolation threshold in composites containing GNP depends on diverse factors, including the filler’s shape and aspect ratio, material processing conditions, and polymer crystallization. To effectively reduce the percolation threshold of a graphite nanoplatelet (GNP) filler in polypropylene, a coating method followed by compression molding at a slow cooling rate is recommended. The GNPs are first surface-modified or coated with a polymer layer to enhance compatibility with the polypropylene matrix. These coated GNPs are then dispersed in a solvent that also dissolves polypropylene, or directly blended with molten polypropylene using high-shear mixing to ensure uniform distribution. The mixture is compression molded under heat and pressure to align the GNPs within the matrix, enhancing electrical and thermal conductivity. Controlled slow cooling after molding helps achieve better crystallization of polypropylene and alignment of GNPs, thus effectively forming an interconnected network and improving the mechanical strength, electrical conductivity, and thermal stability of the resulting composite. This optimized integration method makes these materials suitable for various applications, including electronics, automotive components, and thermal management systems [112]. This approach allows for the effective interfacial interaction between the matrix and filler controlling either physical or chemical interactions, thereby finely tuning the components’ mixing process until the final material’s consolidation.

## 4. In Situ Exfoliation of Graphite Assisted by Polymers

The top-down approach is cost-effective for exfoliating graphite into derivative forms like graphene. Various methods are used in manufacturing to achieve graphite exfoliation for fabricating polymer graphite composites. These include solution mixing, melt blending, and combinations [45,113], significantly reducing the cost frequently associated with graphitic fillers. This approach has raised great attention due to the potential scalability of graphene-based polymer composite material in applications such as EMI shielding, electrodes, resistors, solar cells, packaging, and flame retardancy [46]. This section focuses on techniques like solution blend mixing, melt processing, and ball milling, which can be used for the in situ exfoliation of graphite into derivative forms similar to graphene, leading to the fabrication of graphene-based polymer composites. Table 1 shows a summary of the main advantages and disadvantages of the technique

The advantages of the polymer-assisted graphite exfoliation method are particularly notable due to the economy of the raw materials, including the widely available and commonly used graphite and polymers. Furthermore, the processing of these materials involves only a few steps, significantly simplifying production. This efficiency enables the rapid formation of new multifunctional materials using accessible technologies, offering an optimal balance between performance and cost.

### 4.1. Solution Blend Mixing

Solution blend mixing involves dissolving polymers and fillers to facilitate the wettability and interaction between the components [114]. The exfoliation in solvents has attracted attention for its potential in producing stable and high-concentration graphene dispersions, outperforming pure organic solvent and small molecular weight surfactant methods. This method leverages strong interfacial interactions and steric repulsion to facilitate exfoliation and stabilization. Despite significant advances, challenges remain in fully understanding and optimizing the interfacial interactions and steric repulsion necessary for large-scale graphene production [115]. Li et al. developed a nanocomposite based on GNP using ultrasonication in acetone. First, they expanded the graphite with ultrasound in the solvent. Then, the graphite surface was modified with UV/ozone to obtain hydroxyl, ether, carboxyl, and carbonyl groups, which improved the interfacial interactions with the epoxy matrix. Even though the amount of oxygenated functional groups generated by oxidation was only marginal (e.g., C-OH or C-O-C content on pristine GNP before treatment was 4.9%, and after 70 min of UV/ozone treatment increased to 7.7% only), it has a positive impact due to the extensive surface area of GNP used in the study. Then, 2% of the resulting GNP was mixed with epoxy resin, which was again ultrasonicated to exfoliate, disperse, and stabilize the GNP in the polymer mixture. The procedure reduced the size of the graphite particles intercalated into the GNP. Next, a curing agent was added to crosslink the polymer at 80 °C under low pressure for two hours to obtain the final product. It also received a post-curing treatment at 150 °C for 3 h in an air environment. The consolidated material showed an electrical resistivity of 5.2 × 10^5^ Ω cm and a surface area of 338 m^2^/g [25].

Khanam et al. developed a practical, scalable, and efficient method for producing graphene dispersions in an eco-friendly solution of poly(vinyl alcohol) (PVA) in H_2_O (0.025, 0.05, 0.1, and 0.5 wt%) via exfoliation of graphite using an ultrasonic system. They obtained high-quality graphene flakes with about 1–5 layers and dimensions up to 1.48 nm of width and 0.3 to 4 µm of lateral size from graphite of 20 μm. The PVA-stabilized graphene dispersion was then used as a coating on a paper substrate, solid composite, or thin films. Remarkably, the coating on paper materials showed an excellent electrical conductivity of 6666 S/m and good flexibility without any annealing post-treatment. Besides, this dispersion can be used as an additive material for mixing with other matrices [116] or as a composite to be used in supercapacitor applications to get a graphene flake of 1.68 nm width and 0.5 to 2 µm of lateral size from graphite of 20 µm [81].

Yuan et al. produced lightweight exfoliated graphite (ExG) by mixing with PVA using a hypergravity-induced self-assembly method [117]. This process significantly reduced the graphite size from 85 μm to achieve a mean lateral size of approximately 60.4 μm and a thickness of 33.8 nm. The resulting ExG/PVA composites exhibited outstanding thermal conductivity (2.42 to 8.45 W/m·K), electrical conductivity (8.16 to 230.40 S/m), and high EMI shielding effectiveness (up to 77.51 dB) while maintaining a low density material (0.28 to 0.64 g/cm^3^). These properties make the composites highly effective for thermal and EMI management applications in advanced electronics.

Rathi et al. developed polyvinylidene fluoride (PVDF) composite films filled with exfoliated graphite (ExG) and ionic liquid (IL) for effective electromagnetic interference (EMI) shielding. The study aimed to create lightweight, flexible, and cost-effective shielding materials. Natural graphite flakes (10–20 µm) were used as filler material and mixed with PVDF using a magnetic stirrer, followed by the addition of ionic liquid (IL) and dissolution in an N,N-dimethylformamide (DMF) matrix. Then, films were fabricated and characterized by electronic microscopy for surface morphology, DSC for thermal transition properties, and impedance analysis for dielectric properties. An optimal composition (70% PVDF, 10% Gr, 20% IL) exhibited excellent EMI shielding effectiveness: 35 dB for plane waves, 120 dB for electric fields, and 40 dB for magnetic fields, with an ultimate tensile strength of 3.25 ± 0.2 MPa and 35% elongation. The study highlights that including IL enhances porosity and absorption loss, improving shielding performance while maintaining flexibility and mechanical strength, suitable for coatings on electronic devices [118].

Gümüs et al. fabricated an effective electromagnetic interference (EMI) shielding composite by mixing an epoxy matrix with graphite of 30 µm and carbon black (CB) as fillers. The study highlights the composites’ dielectric properties and shielding performance, which were prepared with various filler ratios (1–7 wt%) and analyzed across a frequency range of 1–14 GHz. The study found that graphite significantly improved EMI shielding efficiency due to its electrical conductivity, which results from its layered structure. The exfoliation of graphite layers facilitates the formation of conductive pathways, thereby improving the absorption and reflection of electromagnetic waves. The findings indicate that graphite-filled composites exhibited slightly higher shielding efficiency (19–21 dB) than carbon black-filled composites (8–17 dB), making them more effective for EMI shielding applications [119].

### 4.2. Melt Processing

Melt processing involves liquefying a solid polymer to reduce its viscosity, allowing it to be mixed with a filler material. It is one of the most important processing methods for thermoplastic and is widely implemented with extrusion and injection molding techniques [120]. Both techniques involve the following sequence of steps: (a) heating and melting the polymer, (b) pumping the polymer into the shaping unit, (c) forming the melt into the required shape and dimensions, and (d) cooling and solidification. In addition, screw extruders heat and melt the polymer. In simplest terms, screw extruders are polymer pumps with the capacity to melt the material with which they are fed. Screw extruders comprise one or two Archimedean screws rotating in a heated barrel [121]. The twin screw can be a good technique for composite manufacture, but to obtain a homogeneous graphene dispersion within the polymer matrix is not a simple task. Segregation and agglomeration of the nanophase are responsible for the lack of improvement in the thermal, mechanical, and electrical properties of unmodified polymer matrix materials. To address this issue, an effective strategy is to modify the surface of the filler to enhance its interaction with the polymer matrix. It has been reported that while larger sizes of nanofillers may reduce mechanical properties, they can increase other functional properties, such as electrical conductivity [122]

**Figure 2:**
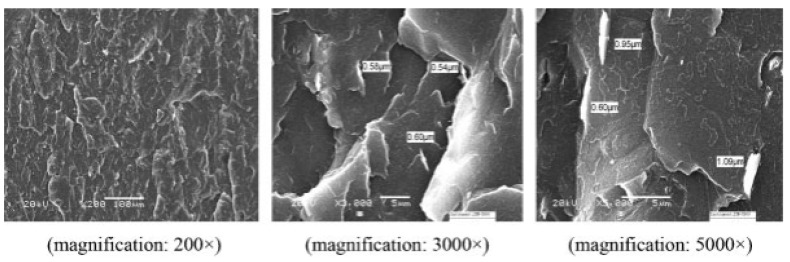
SEM micrographs of rolling intercalation PS with 5% of colloidal graphite [27]. Reproduced with permission from Tu, H.; Polymers for advanced technologies; Published by John/Wiley & Sons Ltd.; 2008.

Tu et al. fabricated a composite material using polystyrene (PS) as the matrix and colloidal graphite as the filler, with an average diameter of 4 µm. The authors modified the graphite surface with silane-coupling agents in an alcohol solution. Then, the graphite suspension was ultrasonicated, filtered, and dried. The functionalized graphite was used to prepare a composite with PS by means of different procedures. The first method, rolling intercalation, consisted of adding PS granules and the functionalized graphite into a high-speed mixer and then to a twin-roller mixer at 120 °C. The final mixture was molded by compression molding at 190 °C. The second method used was solvent intercalation. First, the functionalized graphite was dispersed in THF by ultrasonication. The PS granules were dissolved in the THF-graphite suspension at 50 °C for 8 h and then dried at low pressure for 24 hr. The final mixture was molded by compression molding at 190 °C. Finally, the authors used pan-milling intercalation. In this procedure, the mixture of PS granules and the functionalized graphite was milled by three-dimensional grinding disks. The polymer/filler mixture was compression molded at 190 °C. The authors obtained a composite with a thermal conductivity of 1.95 W/m·K when the polymer was filled with 34 vol% of functional graphite. The exfoliation of the material was analyzed with wide-angle X-ray scattering (WAXD). The authors found that the diffraction peak intensity of graphite in the composites decreased remarkably in the order of rolling intercalation, solvent intercalation, and pan-milling intercalation. This indicates an increasing degree of layer exfoliation of the graphite. The rolling intercalation method effectively disintegrated the graphite agglomerates, resulting in a well-dispersed system with graphite agglomerates measuring 0.5 to 1.0 µm in diameter. It was concluded that this method provides sufficient stress to disintegrate the agglomerates and promote the homogeneous dispersion of the graphite platelets, as shown in Figure 2 [27].

Steurer, P. et al., fabricated a polymeric composite with thermally reduced graphite oxides (TRGO), and the polymers polyamide 6 (PA6), polycarbonate (PC), polystyrene-co-acrylonitrile (SAM), and isotactic polypropylene (iPP) using melt extrusion as their technique with working temperatures of 250 °C (PA6), 280 °C (PC), 210 °C (SAM), and 210 °C (iPP). These composites showed reasonably good electrical resistivity compared to MWCNTs and carbon black in the range of 1.0 × 10^3^ to 2.7 × 10^9^ Ω·× cm [123].

**Figure 3:**
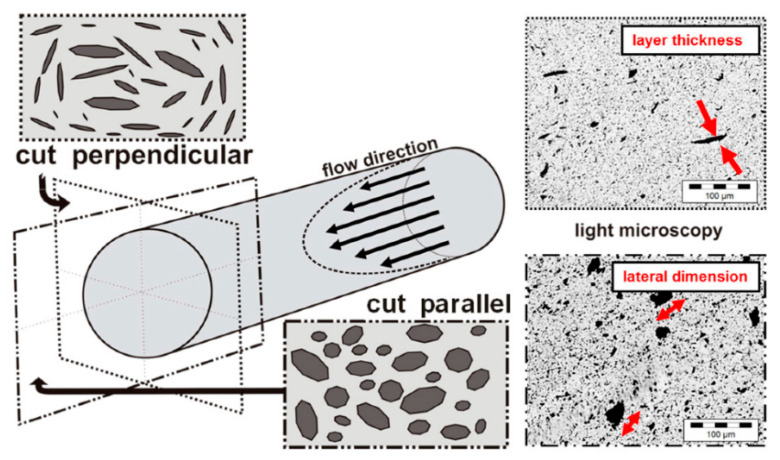
Orientation of the graphite platelet structures in extruded strands: (left) schematic figure of platelet orientation along strand flow direction by extrusion out of the die; and (right) transmission light microscopy pictures of samples cut perpendicular to the strand direction (shows mainly the layer thickness) and cut parallel to the long-axis of the strand (shows the lateral dimension of visible GNP structures), here shown for 1 wt% Graphene nanopowder AO-3 in PC [124]. Reproduced with permission from Pötschke, P.; Materials; Published by MDPI; 2017.

Müller, M.T., et al. [124] used polycarbonate (PC) as the matrix material and incorporated graphite nanoplatelets (GNPs) of various sizes, ranging from 2 µm to 305 µm. They fabricated the composites using a twin-screw micro-compounder under varying conditions, including mixing speeds from 50 to 250 rpm, melt temperatures from 240 °C to 320 °C, and processing times of 5 to 30 min. The resulting composites were molded to produce samples with different degrees of filler distribution. Increasing shear stress, calculated as specific mechanical energy (ranging from 1 to 9 kWh/kg), reduced the lateral dimensions of the fillers (to a range of 2.5 to 30 µm). The composite containing 10 wt% of filler exhibited an electrical conductivity of 10^−4^ S cm^−1^ and a thermal conductivity of 0.71 W/m·K, which is 196% higher than that of pure polycarbonate. Additionally, this composite demonstrated a 160% increase of the relative storage modulus G’. GNP dispersion was characterized by light transmission microscopy analysis on thin sections of extruded strands. The anisotropy of the GNP structures causes orientation during extrusion, with different area ratios detected depending on the direction. In the perpendicular direction, the thickness of the particles is mainly observed, while in the parallel direction, the lateral dimension is seen. Thus, larger particle sizes are visible in the parallel direction, resulting in a larger area ratio. To fully exploit the exfoliated graphite nanoplates, this progress is best observed in images cut perpendicular to the strand. In general, the dispersion of different GNP types varied similarly with melting temperature, screw speed, and mixing time. The increase in shear stress reduces the lateral dimensions of the GNP structures, decreasing their aspect ratio (Figure 3).

**Figure 4:**
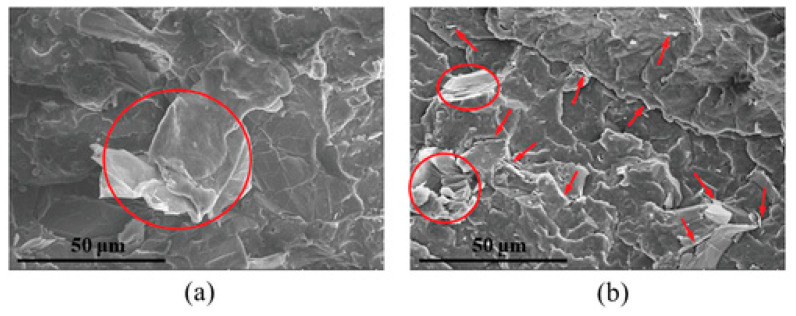
SEM images of (**a**) PVDF/expanded graphite composite fabricated without water (P-EG) and (**b**) PVDF/expanded graphite composite fabricated with water (P-EG-W) samples [91]. Reproduced with permission from Tong, J.; Macromolecular materials and engineering; Published by Wiley-VCH Verlag GMBH & Co.; 2020.

Tong, J., et al., [91] fabricated a composite using poly(vinylidene fluoride) (PVDF) as the matrix and expanded graphite (EG) as the filler. The composite was prepared using melt mixing in a twin-screw extruder. The processing conditions included 4 wt% of filler, a screw speed of 100 rpm, and a barrel temperature profile that started at 180 °C, increased to 235 °C, and then decreased to 200 °C. During the process, water was injected into the middle section of the extruder as a plasticizer agent and subsequently removed using a vacuum pump located in the last section of the extruder before the nozzle. The mixture underwent a pelletizing step, followed by drying at low pressure, and finally, compression molding for shaping. The objective was to compare the properties of the material processed with water to those of the material processed without water. The water-assisted process resulted in better exfoliation and dispersion of the filler, achieving a filler size of less than 25 µm. Figure 4a,b shows the scanning electron microscopy images of the PVDF/expanded graphite composite fabricated without water (P-EG) and PVDF/expanded graphite composite fabricated with water (P-EG-W) samples, respectively. It can be seen that the expanded graphite exhibits poor exfoliation and dispersion in the PVDF matrix, with large aggregates (up to 50 μm in diameter) found in the P-EG sample, as indicated by the circles in Figure 4a. With the injection of water, the P-EG-W sample shows better-exfoliated and dispersed EG. As illustrated in Figure 4b, the aggregates are exfoliated into smaller sizes of less than 25 μm in diameter, and some few-layer graphite platelets are achieved, as indicated by the arrows in Figure 4b (Figure 4). The composites (P-EG-W) demonstrated an electrical conductivity of 3 × 10^−11^ S/m and a thermal conductivity of 0.395 W/m·K, which is 101.5% higher than the virgin matrix. The samples exhibited an increased Young’s modulus and higher tensile strength, with improvements of 64.5% and 28.1%, respectively, compared to the virgin matrix.

**Figure 5:**
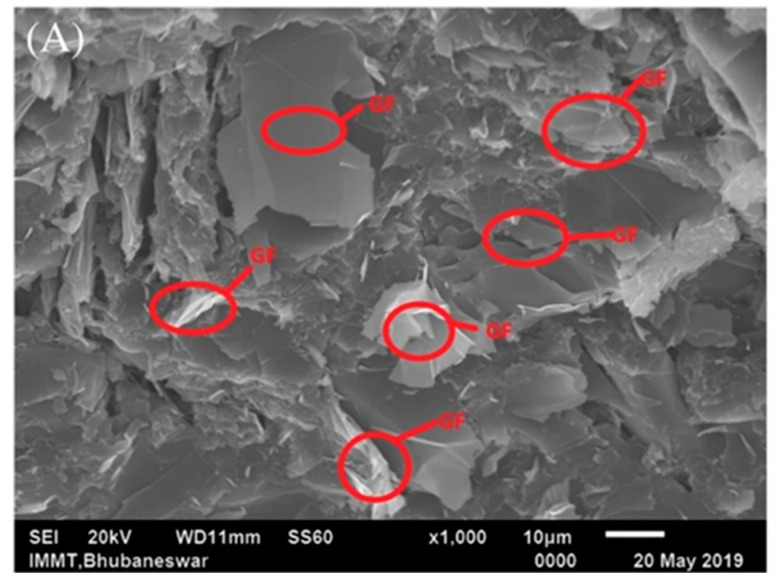
FESEM images of (**A**) PCGF-30 [87]. Reproduced with permission from Pradhan, S.S.; Polymer composites; Published by John/Wiley & Sons Ltd.; 2021.

Pradhan, S.S. et al., produced a nanocomposite using polycarbonate as the matrix and graphite flakes (GF) with a particle size of 44 µm as fillers. They employed melt mixing in a screw mixer at 60 rpm and 240 °C for 12 min. The samples were prepared using a mini-injection jet with a pressure of 8 bar and a mold temperature of 40 °C. This method resulted in the formation of small graphite flake structures due to the combination of high pressure and temperature during melt mixing. The good dispersion of the filler improved the tensile modulus of the composite. The presence of small GF particles in the matrix ensures better dispersion within the polymer, which enhances the tensile modulus of the composite. Additionally, at high filler loading, the GF particles form a bridged network, leading to improved thermal conductivity. Figure 5 shows that at high filler loading, the GF particles tend to agglomerate, which may contribute to the decreased strain values observed during mechanical testing (Figure 5). However, at high filler loadings, the distribution of GF became irregular within the matrix, producing a “ridge-rock” morphology, which increased the rigidity of the composite. The composite’s tensile and impact strength ensured its moldability. The samples exhibited an electrical conductivity of 0.3  ×  10^−2^ S/cm with 20 wt% of filler and an EMI shielding effectiveness of approximately 35 dB with 30 wt% of filler [87].

Kaczor, D. et al., demonstrated the production of a composite using industrial grade graphite as fillers, with sizes ranging from 6 µm to 150 µm, and poly(lactic acid) (PLA) as the matrix. The material was prepared by melt mixing using a Brabender, with a weight ratio of 1:3 filler to polymer. The processing conditions were 50 rpm at 190 °C for 2 min. This method was effective in decreasing the size of the filler [82].

Yin, X. et al. used in situ melt mixing to expand graphite. Initially, they intercalated the filler using PVA (concentration 3 wt%) in an aqueous solution. The mixture was kept at 70 °C and stirred continuously for 1 h in the constant temperature. Subsequently, the mixture was processed with low-density polyethylene (LDPE) as the matrix in a Brabender mixer set at 200 °C and 60 rpm for 8 min, incorporating 5 wt% of intercalated graphite. This method facilitated in situ expansion of the graphite during melt processing due to the expansion of water in the form of gas intercalated in the graphite. The resulting material exhibited significant exfoliation, as indicated by the decrease intensity of the (002) diffraction peak of XRD pattern, and demonstrated a final thermal conductivity of 0.715 W/m·K [92].

Baptista, R., et al., evaluated the presence of graphite platelets in an epoxy-carbon matrix with graphite having an average diameter of 10.5 µm, at contents ranging from 0 to 20 wt%. The mechanical properties of composites consisting of epoxy resin reinforced with graphite platelets were also compared with those of hybrids consisting of graphite/epoxy resin reinforced with carbon fiber. To prepare the materials, the researchers first heated the resin in an oven at 40 °C for 12 h to decrease the epoxy’s viscosity. Subsequently, graphite platelets were added to the resin, and the resin/graphite mixture was hand-mixed at room temperature for 5 min. Raman spectroscopy was employed to analyze the chemical bonding between the filler and the matrix. The peak areas of the D and G bands for graphite and the graphite-epoxy composite were compared. A decrease in the D to G peak area ratio was observed, indicating a reduction in crystalline lattice defects when the graphite bonded to the polymer matrix. Additional evidence was obtained by comparing the Raman spectrum of the neat resin with that of the graphite/epoxy composite. A notable decrease in the intensity of the resin peaks at 1109, 1186, and 1609 cm^−1^ was reported, suggesting that the presence of graphite renders the corresponding epoxy vibrations inactive. This provides further evidence of the chemical bonding between the resin and graphite. The simple mixing method highlights the significance of the interfacial interactions between the matrix and the filler in creating an effective composite. Graphite/epoxy composites reinforced with carbon fiber demonstrated superior mechanical and wear performance compared to conventional carbon fiber-reinforced epoxy composites [26].

Hendrix, J. et al., used natural graphite flakes of 250 µm with polyamide 6, 6 (PA66) to prepare a polymer composite. The composite was prepared using a high-shear mixer to provide an elongational flow. The blend was mixed at 276 °C with a shear strain rate of 2876 s^−1^ under an argon atmosphere to reduce polymer degradation. The authors successfully exfoliated the graphite layers within the composite in only 30 min, reducing the graphite’s initial size by up to 76%. The positive outcomes of this research can be attributed to the high shear forces generated by the high-speed elongational flow method and the effective diffusion of PA66 into the graphite layers. However, further investigations are necessary to elucidate the interfacial properties between PA66 and graphite, as well as the specific bonding mechanisms involved [88].

Wang et al. fabricated a polypropylene (PP)/graphite intercalation compound via melt mixing at a rotor speed of 100 rpm. Three different temperatures were used to generate the composites: 180 °C (PG180), 200 °C (PG200), and 220 °C (PG220). The initial intercalated graphite had a particle size of 300 µm, and composite samples were prepared with a graphite content of 10 wt%. The graphite structure exhibited a smaller size and better dispersion in the PP matrix in samples prepared at 220 °C (PG220) compared to those prepared at lower temperatures (PG180 and PG200). This improvement is attributed to the higher expansion of graphite due to the release of gases (CO_2_ and H_2_O) during the process. The graphite expansion enhances the intercalation of PP chains into the graphite layers, and the higher temperature reduces the viscosity of PP, facilitating the intercalation activity. The PG220 samples demonstrated a dielectric constant of 1.3 × 10^8^ at 10^3^ Hz, which is approximately six and five orders of magnitude higher than PG180 and PG200, respectively. Additionally, the thermal conductivity of PG220 increased by 61.5% (0.63 W/m·K) compared to neat PP. PG220 also exhibited a Young’s modulus approximately 50.7%, 21.2%, and 10.0% higher than that of the neat PP, PG180, and PG200 samples, respectively. However, the elongation at break of the PG220 sample substantially decreased to about 5.5% compared to neat PP. This reduction indicates poor interfacial adhesion between the PP and graphite, due to poor wettability, which impedes effective stress transfer across the PP matrix-graphite interface [29] This highlights the critical importance of optimizing both the employed methodology and the interfacial interactions between the components in composite materials.

An innovative method for large-scale preparation of natural rubber/expanded graphite (NR/EG) composites with enhanced thermal conductivity involves using a dry ice explosion. This technique facilitates the exfoliation and dispersion of expanded graphite within the natural rubber matrix. The process begins with pre-mixing the rubber with a vulcanization system, followed by the addition of graphite and dry ice. The rapid sublimation of dry ice creates an explosive shock wave that lowers the material’s temperature, enhances shear forces, and promotes effective dispersion. The resulting composites, vulcanized at 180 °C and 10 MPa, exhibited significant improvements in thermal conductivity, reaching 2.317 W/(m⋅K) at 30 wt% EG content, compared to 1.206 W/(m⋅K) without dry ice, representing a 92.1% improvement. Additionally, the mechanical properties were enhanced, with a 33.7% increase of tensile strength and a 9.2% increase of elongation at break. This method provides an efficient, environmentally friendly, and scalable approach to producing high thermal conductivity composites without the use of chemical solvents, with potential applications in thermal interface materials for electronic packaging [89].

A lightweight, flexible, and cost-effective electromagnetic interference (EMI) shielding material was developed using exfoliated graphite nanoplatelets (xGnP), filled ethylene vinyl acetate (EVA), and ethylene-octene copolymer (EOC) blend composites. These composites were created via a melt mixing method and evaluated for their EMI shielding effectiveness in the S-band (2–4 GHz) frequency range. The inclusion of xGnP significantly improved the EMI shielding effectiveness, achieving a maximum total EMI SE value of −67.63 dB at 30 wt% xGnP loading, primarily due to absorption mechanisms. The composite also exhibited enhanced electrical conductivity, with an AC conductivity of 455.8 S/m and a DC conductivity of 5.3 × 10^−8^ S/m for the 30 wt% xGnP EVA/EOC blend. The exfoliation of graphite nanoplatelets from 1–2 μm to an average thickness of 2 nm, with processing likely reducing their effective size and enhancing dispersion within the matrix, was indicated by changes in crystallinity and morphology observed through SEM and XRD analyses [90].

Wei, B et al. reported a facile, low-cost, and scalable method to fabricate an enhanced 3D expanded graphite (EG) network with excellent EMI shielding performance in linear low-density polyethylene in LLDPE composites. By pre-melt blending EG (with an average particle size of 150 µm) with stearic acid and polyethylene wax, followed by powder mixing and thermal molding, the study achieved a significant reduction in interfacial thermal resistance. The resulting composites exhibited ultra-high thermal conductivity of up to 19.6 W/m·K and excellent electromagnetic interference (EMI) shielding effectiveness of 52.4 dB, with electrical conductivity reaching up to 4000 S/m. This approach provides a promising solution for thermal management and EMI shielding in electronic devices, demonstrating high cooling efficiency and robust thermal transfer capabilities [86]

A method to enhance thermal conductivity and electromagnetic interference (EMI) shielding in polymer composites involves regulating the orientation and exfoliation of 23 µm graphite flakes (GF) using hollow glass microspheres (HGμS, soda-lime borosilicate glass particles). The 3D GF network formation relies on the coordination of GF and HGμS particles, where the former is confined in the interstitial regions of the close-packing structures of HGμS. Remarkably, the entrapment inhibits the in-plane orientation of FG along the flow direction during melt processing and facilitates its exfoliation under the external forces applied by a torque rheometer. The combination of thermoplastic polyurethane (TPU), GF, and HGμS forms a 3D interconnected network, achieving in-plane and through-plane thermal conductivities of 20.54 W/m·K and 6.55 W/m·K, respectively, with 30 vol% GF, and an EMI shielding effectiveness of 69 dB at 1 mm thickness. These materials and processes resulted in lightweight, highly conductive composites suitable for advanced electronics applications [125].

A solvent-free, scalable method to fabricate asphalt/graphite nanoplatelet (GNP) composites uses a three-roll mill (TRM) for in situ exfoliation of graphite in an asphalt melt. The exfoliation process significantly reduces the size of graphite particles from 78 µm to a lateral size of 1–4 µm and thickness to 20–30 nm, enhancing dispersion in the asphalt matrix. This reduction in particle size leads to significant improvements in thermal conductivity and mechanical properties. Notably, at 25 vol% loading, the thermal conductivity of the asphalt/GNP composite reached 1.95 W/m·K, i.e., a 114% increase compared to conventional asphalt/commercial GNP (c-GNP) composites. The method also improved heat resistance and mechanical properties, making these composites suitable as high-performance thermal interface materials (TIMs) [83].

### 4.3. Ball Milling

Ball milling is an effective mechanical exfoliation technique for the scalable production of graphene-like nanostructures. This method involves a chamber containing primarily steel balls and the material to be ground. As the chamber rotates, the balls collide with each other, the walls, and the sample, generating impact force and shear stress on the graphite structures. Consequently, the graphite is fragmented, producing graphene-like nanostructures. Despite the probability of generating graphite with suboptimal morphologies and limited surface area, the ball milling technique is widely used in industry due to its low cost and scalability [50]. Ball milling can be utilized to produce nanocrystals by harnessing the energy of the mill, along with the deformation and fracturing of powder particles, to synthesize new materials. However, a critical challenge remains in consolidating milled powders into full-density bulk materials with practical shapes and adequate sizes without significant structural alterations. This difficulty arises due to the tendency of milled powders to exhibit changes in crystallinity and phase composition during the consolidation process, which can impact the mechanical and functional properties of the final bulk material [70]. Depending on the ball configuration, material, and barrel, ball milling can be used for mechanical coatings [71]. Additionally, ball milling can be conducted under gas pressure for the exfoliation and functionalization of graphite. Through mechanochemical processes, the hydroxylation or carboxylation of graphite sheets can be achieved, enhancing their interfacial interaction with the matrix. This functionalization improves the compatibility and bonding between the graphite and the surrounding material, leading to better dispersion and enhanced properties in the resulting composite [126].

Generally, ball milling aims to reduce particle size. However, it can also be applied to processes such as amorphization, particle size growth, shape control, agglomeration, solid-state reactions through mechanical alloying, mechano-fusion, property modification in specific materials, manufacturing advanced minerals, producing high-performance cementitious materials, and preparing composites [127]. This technique can also be used to generate metal alloys mechanically through the repeated processes of cold welding, fracturing, and rewelding of metallic powder particles in a high-energy ball mill. This positions it as an effective method for producing metastable and advanced materials with significant potential for widespread applications [128]. The synthesis of nanocomposites via mechanochemical milling for energy storage applications requires the optimization of milling parameters such as mill geometry, the circumferential velocity of the stirrer, ball size, milling time, and energy input. The simplicity, cost-effectiveness, and scalability of this method make it advantageous for producing various nanocomposites with controlled properties. These nanocomposites show promising potential in electrochemical and hydrogen storage applications due to their enhanced mechanical properties and suitability for large-scale production [129].

As previously mentioned, the impact and friction between the balls and the material result in size reduction and mixing. However, ball milling can also lead to particle agglomeration and growth. High-energy impacts can cause particles to fracture but also lead to smaller particles colliding and sticking together, resulting in agglomeration. Shearing can flatten particles and create surfaces with high surface energy, promoting agglomeration as particles stick together to minimize surface energy. Milling increases surface area, which raises surface energy and drives particles to agglomerate to reduce the overall energy. Surface chemistry also plays a role—chemically active surfaces or functional groups can form stronger agglomerates. Mechanical energy from milling can convert to heat, raising the temperature and enhancing diffusion and sintering, leading to particle growth. An inappropriate ball-to-powder ratio can cause excessive collision energy, making particles stick together rather than breaking down. Higher speeds and longer milling times increase agglomeration due to continuous collisions and prolonged exposure to mechanical forces. Material properties also contribute to agglomeration; ductile materials are more prone to flattening and agglomeration, while brittle materials may fragment easily but still form agglomerates through surface interactions. Wide size distributions can also lead to agglomeration as smaller particles fill gaps between larger particles.

Nevertheless, there are several strategies to mitigate agglomeration. Optimizing the ball-to-powder ratio, milling speed, and milling time is crucial for balancing size reduction and minimizing agglomeration. Lowering temperatures can reduce thermal effects that promote agglomeration. Additives can reduce surface energy or prevent particles from sticking together. Post-milling treatments, such as sonication or the use of dispersing agents, can help break up agglomerates formed during milling.

Overall, ball milling can lead to particle agglomeration and growth due to high surface energy, mechanical forces, temperature increases, and material properties. Optimizing milling conditions and using appropriate additives can help mitigate these effects and achieve finer, more uniform particle distributions.

Dong et al. fabricated nanocomposites composed of graphene/polyvinylidene fluoride and graphene/thermoplastic polyurethanes for electromagnetic shielding applications using industrial grade expandable graphite (297 µm). They prepared the composites by matching the viscoelastic properties of the polymer with the impact shear field of high-energy ball milling. They obtained graphene structures with 4–7 layers when the graphite content was 6 wt%. These composites exhibited conductivity ranging from 0.41 S/m to 1.01 S/m and an electromagnetic shielding effectiveness of 15.8 to 21.4 dB [28].

**Figure 6:**
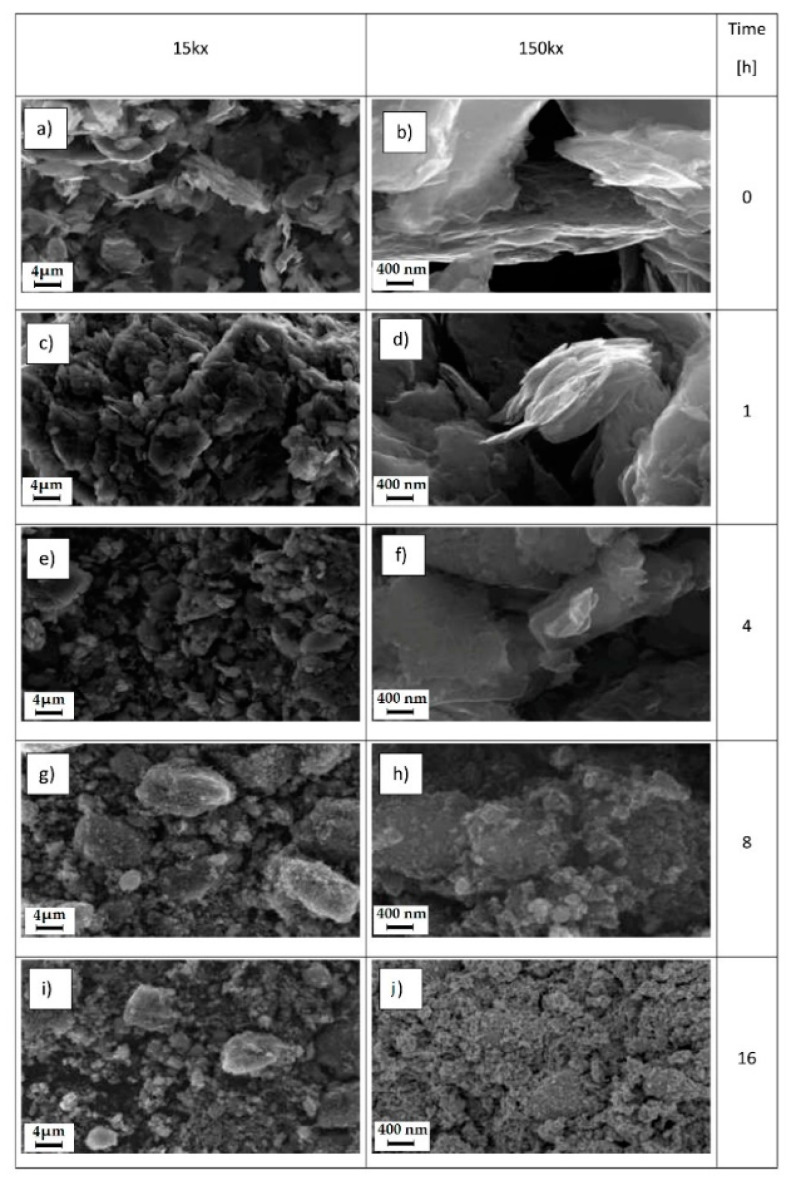
SEM images of graphite at magnifications of 15 and 150 Kx at different ball milling times: (**a**,**b**) 0 h; (**c**,**d**) 1 h; (**e**,**f**) 4 h; (**g**,**h**) 8 h; (**i**,**j**) 16 h [130]. Reproduced with permission from Visco, A.; Polymers; Published by MDPI; 2021.

Visco, A. et al. exfoliated graphene using a ball milling operating at a frequency of 20 Hz for different durations, ranging from 1 to 16 h. They successfully reduced the graphite size from 340 nm to clusters smaller than 190 nm. The resulting graphite was then utilized to fabricate a composite with high-density polyethylene (HDPE). Using a Brabender machine at 180 °C for 15 min with a filler amount of 0.3 wt%, the composite material exhibited significant improvements in stiffness and yield strength. Additionally, the nanocomposites demonstrated a progressive increase in wear resistance, with the maximum improvement reaching approximately 65%. However, the enhancement in thermal resistance was marginal. SEM morphological investigations were performed on pure and ball-milled graphite at various time intervals. Figure 6a,b (0 h) and Figure 6c,d (1 h) show large graphite planes that remained nearly with the same width or slightly reduced after one hour of ball milling. These images suggest that the mechanical treatment did not significantly alter the graphite structure after one hour, as the graphitic planes remained wide and extended (greater than 200 nm). This pattern continued after four hours of treatment. Noticeable fragmentation of the graphene planes required extended mechanical treatment—at least 8 h (Figure 6g,h) or ideally 16 h (Figure 6i,l). After 16 h, significant fragmentation into very small particles was observed, with large graphitic layers breaking down into clusters smaller than 200 nm (Figure 6) [130].

Navik, R. et al. developed an eco-friendly and industrially viable methodology to fabricate polypropylene (PP) and few-layer graphene (FLG) composites by combining planetary ball milling and supercritical CO_2_ (scCO_2_). The use of scCO_2_ was strategic because it acts as a polymer plasticizer and enhances FLG/polymer compatibility by decreasing interfacial tension, resulting in homogeneous dispersion of the filler within the polymer matrix. Supercritical CO_2_ is particularly advantageous due to its zero-surface tension, gas-like diffusivity, low viscosity, low cost, and nontoxicity. This approach enables efficient in situ exfoliation of graphite, initially sized at 7 µm, into 1–6-layer graphene and improves interfacial compatibility without the use of organic solvents or toxic auxiliaries.

The resulting composites exhibit significant improvements in thermal conductivity, reaching up to 6.15 W/m·K, and electrical conductivity, up to 4.6 × 10^−2^ S/m. Mechanical properties were also notably enhanced. This technique offers a sustainable process with potential for advanced electronic applications, achieving efficient exfoliation and homogeneous dispersion of graphene within the polymer matrix [84].

The same authors developed a sustainable and scalable method for producing polyamide-6 (PA-6) composites with few-layer graphene (FLG) using mechanochemistry combined with scCO_2_ technology. This process efficiently exfoliates bulk graphite of 7 µm into FLG (≈10 layers), which is uniformly coated onto PA-6 particles. The resulting composite films, containing only 3 wt% FLG, exhibit impressive in-plane electrical conductivity (4.59 S/m), through-plane electrical conductivity (29.59 S/m), EMI shielding effectiveness (41.8 dB), and thermal conductivities (in-plane: 1.78 W/m·K, through-plane: 2.14 W/m·K) [85].

**Summary of Key Findings in Graphite Exfoliation and Nanocomposite Engineering.**

**Figure 7:**
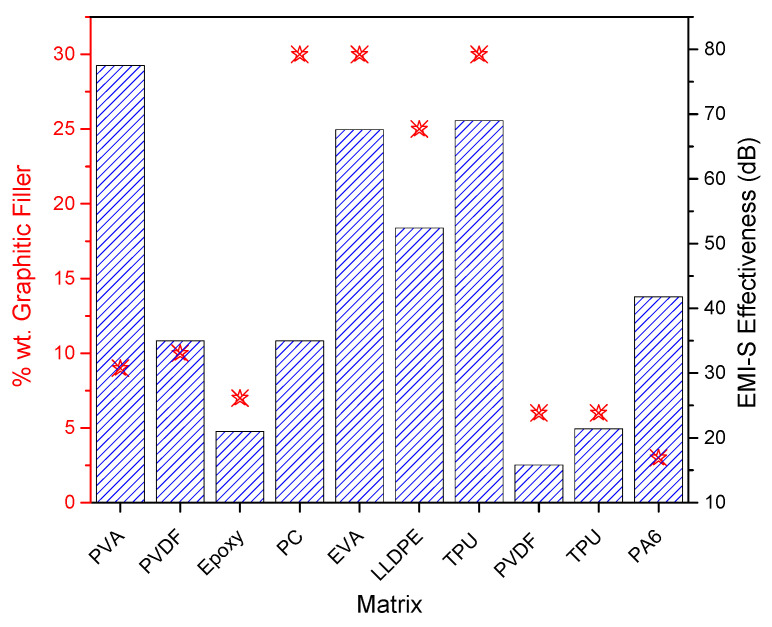
EMI-S effectiveness (dB) and wt% filler in different matrix.

**Figure 8:**
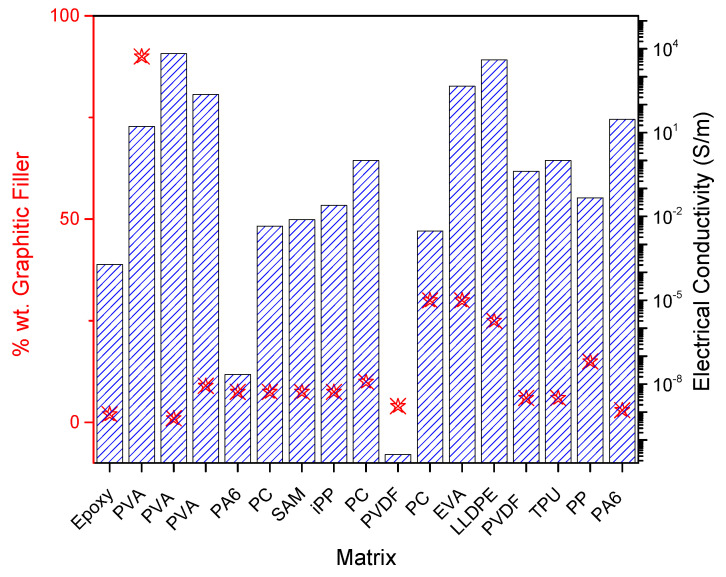
Thermal conductivity and wt% filler in different matrix, conductive nanocomposite.

**Figure 9:**
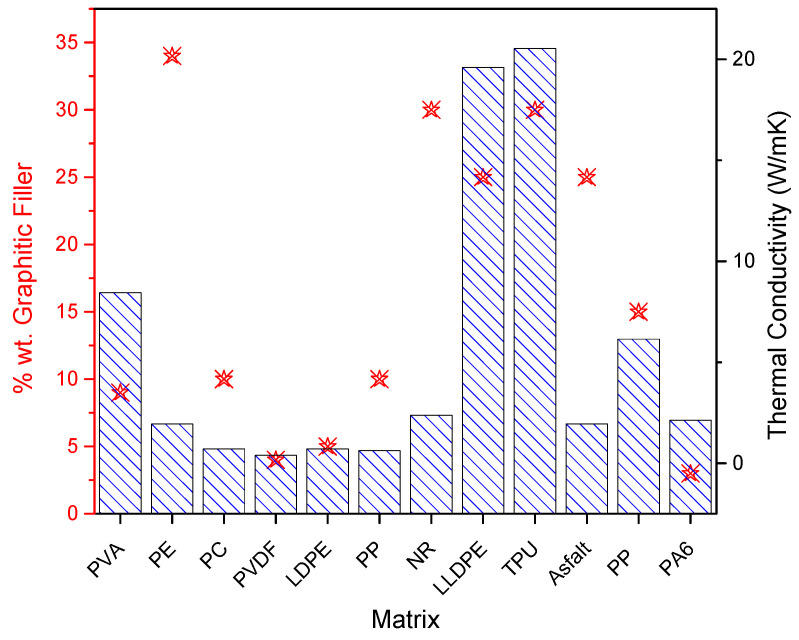
Thermal conductivity and wt% filler in different matrices, conductive nanocomposite.

Table 2 summarizes the application of various composites fabricated using diverse polymer matrices and graphitic structures, employing the state-of-the-art engineering techniques covered in this review. Moreover, Figure 7, Figure 8, and Figure 9 provide a detailed analysis of the impact of different percentages of graphite filler in the matrix on the resulting electromagnetic interference (EMI) shielding effectiveness, electrical conductivity, and thermal conductivity of the polymer composites, respectively. These analyses underscore the critical role of graphite filler content in determining the functional properties of polymer composites. The state-of-the-art techniques employed in the fabrication processes, such as ball milling, scCO_2_ treatment, and various mixing methodologies, ensure the effective dispersion and integration of graphite within the polymer matrices. Consequently, the engineered composites exhibit optimized mechanical, electrical, and thermal properties suitable for advanced applications in electronics, thermal management, and EMI shielding.

During exfoliation, the chemical and physical processes at the interface indicate that specific defects and functional groups on the graphite surface, such as hydroxyls, carbonyls, and carboxylic acids, play a crucial role in overcoming the van der Waals interlayer forces. These functional groups facilitate the intercalation of polymer chains, thereby aiding in the exfoliation process. The steric effect of the polymer significantly contributes to exfoliation. The polymer matrix interacts with the graphite surface primarily through weak van der Waals forces, which help overcome the internal interactions within the graphite, especially in the case of polymers like polyolefins. In some instances, exfoliation is driven by hydrogen bonds formed between the matrix and the filler, as observed with polyamides and polyols [79,86]. Polymers with aromatic groups, such as polycarbonates and polystyrenes, also promote exfoliation through π-π stacking, allowing additional interactions with the surface. Moreover, covalent bonds can form between the matrix and the filler, as seen with epoxies on oxidized surfaces [25]. Together, steric repulsion forces, van der Waals forces, hydrogen bonds, π-π stacking, and covalent bonds, along with shear forces, significantly contribute to the exfoliation, dispersion, and stabilization of exfoliated structures. These interactions prevent aggregation and generate new functional polymeric composite materials.

**Summary of enhanced properties of nanocomposites containing exfoliated graphene derivatives.**

Figure 7 illustrates the variability in the percentage of graphitic filler between different polymers, revealing that higher percentages of filler do provide greater EMI shielding effectiveness, but this relationship does not always directly correlate with greater EMI shielding effectiveness. For example, while polymers such as PVA with a low percentage of graphitic loading exhibit high EMI shielding effectiveness, others such as PC with a high percentage of loading show lower effectiveness. This indicates that the effectiveness of EMI shielding is influenced not only by the amount of graphitic filler but also by the physical and chemical interactions between the filler and the polymer matrix. Factors such as graphite dispersion and graphite particle orientation play a critical role in determining the ultimate effectiveness of EMI shielding in these composites.

In Figure 8, the variability in the percentage of graphitic filler between different polymers is notable and does not show a direct and consistent correlation with the electrical conductivity of the matrices. This indicates that other factors, in addition to the graphite content, influence electrical conductivity. This suggests that the electrical conductivity of these polymeric matrices depends not only on the amount of graphite they contain but also on how the graphite is distributed and interacts within the matrix. Factors such as the dispersion of graphite and the orientation of graphite particles within the matrix forming percolation networks can significantly impact electrical conductivity. This underlines the importance of optimizing these variables to improve the electrical conduction properties of graphite-based composites.

In Figure 9, considerable variability in the percentage of graphite filler among different polymers is observed, and this variability does not always directly correlate with an increase in thermal conductivity. This indicates that thermal conductivity in these composites is influenced not only by the amount of graphite incorporated but also by how the graphite is integrated within the polymer matrix. Factors like these play critical roles in determining the final thermal conductivity of these composites. This highlights the importance of optimizing not only the quantity but also the quality of graphite integration to improve the thermal conductivity properties of graphite-based composites.

## 5. Conclusions and Outlook

This document underscores a variety of techniques for exfoliating graphite assisted by polymers, including solution blend mixing, melt processing, and ball milling. These approaches offer a novel perspective on fabricating multifunctional nanomaterials with fewer steps, which is particularly appealing for the industry. It should be noted that the percolative structure within the bulk of the materials determines the selected methodology, thus affecting the final material and its applicability in different processing scenarios, such as spray coating versus compression molding.

The most notable feature of these methods of nanocomposite production is the avoidance of the cumbersome steps typically used in conventional techniques of graphene production. These innovative approaches not only decrease the number of steps involved in generating the material, leading to reduced manufacturing costs, but also enhance the shear force in the mixture. This, combined with the interfacial interaction with the polymer matrix, promotes the exfoliation of the graphite filler, which is then dispersed and stabilized within the polymeric matrix as nanostructures. Exfoliated graphite embedded in polymer matrices enhances properties like thermal and electrical conductivity and EMI shielding effectiveness. The exfoliation of graphite into nanostructures, particularly when assisted by polymers, is a promising yet complex approach in material science, especially for developing multifunctional nanocomposites. This method leverages the low cost and sustainability of graphite but encounters significant challenges due to the intrinsic properties of graphite and the interactions within composite systems.

The choice of polymeric matrix and the amount of filler can be tailored depending on the end application. A precise selection can improve electrical and thermal conductivities, increase the matrix’s Young’s modulus, and enhance electromagnetic interference shielding and flame retardancy. The most commonly utilized techniques for these purposes are Brabender mixing and ball milling, both of which are scalable and readily available for industrial use. The graphite exfoliated into nanostructures generated by these methods, particularly through polymer-assisted methods, offers a cost-effective and sustainable approach to the fabrication of multifunctional nanocomposites. This has substantial implications for industries seeking scalable solutions.

However, the use of graphite as a filler presents challenges such as limited control over the formation of percolative structures and the polydispersity of the formed structures. Additionally, graphite fillers generally exhibit lower electrical and thermal conductivities compared to other carbonaceous fillers like graphene and carbon nanotubes. Despite these drawbacks, graphites´ low cost and adaptability for mass production make it an attractive option for large-scale applications, particularly in areas requiring cost-efficient materials with moderate performance enhancements, such as in coatings and EMI shielding for electronics.

In summary, while the use of graphite as a filler presents specific challenges, the evolving techniques and strategic integration of graphite into polymer matrices continue to hold substantial promise for the development of advanced materials that balance cost, functionality, and performance across various industrial applications. This synergy between cost-efficiency and functional enhancement through improved manufacturing and composite design represents a key area for future research and industrial application.

Despite the advancements reported earlier, certain areas remain relatively unexplored and hold potential for future research and development. The use of bio-based or green solvents and exfoliation agents derived from natural sources could offer environmentally friendly alternatives to traditional chemical exfoliation methods, reducing the environmental impact and providing safer working conditions. Combining multiple exfoliation techniques in a synergistic manner could enhance the efficiency and quality of exfoliated graphite, leading to the production of high-quality graphene with tailored properties for specific applications. Using microwave irradiation to exfoliate graphite could offer a rapid and energy-efficient method for producing graphene, reducing processing times and energy consumption. Exploring novel surface functionalization techniques that improve the compatibility of exfoliated graphite with various polymer matrices could lead to better dispersion and stability of graphene within polymers. Developing polymer composites with self-healing capabilities that incorporate exfoliated graphite or graphene could find applications in areas requiring long-term durability and reliability. Using electrospinning techniques to fabricate polymer-graphene nanofiber composites could create highly uniform and continuous nanofibers with enhanced mechanical and electrical properties.

Exploring these areas could lead to breakthroughs in the production and application of graphene and graphite-based polymer composites, driving innovation and expanding their use in various high-tech industries.

## Figures and Tables

**Table 1:** Advantages and disadvantages of the processing techniques.

	Advantages	Disadvantages
Solution blend mixing	Scalable process. Good dispersion High quality	Use of solvents Limited polymers
Melt processing	Scalable process. Occurs in a single processing step The high processing temperatures allow the expansion of gases High compatibility with thermoplastics High compatibility with other processing techniques such as injection or compression molding	High energy costs Polydisperse
Ball milling	Scalable process Occurs in a single processing step Blend Versatility Low equipment cost	High polydispersity Increase defects High processing times

**Table 2:** Summary of Key Findings in Graphite Exfoliation.

Matrix	Filler (wt%)	Size before Exfoliation (µm)	Size after Exfoliation	EMI-S (dB)	Electrical Conductivity (S m^−1^)	Thermal Conductivity (Wm^−1^ K^−1^)	Application	Type	Ref.
Epoxy	Intercalated graphite 2%	-	GNP		1.9 ×10^−4^			Solution blend mix	[25]
PVA	Graphite 90%	20	1.48 nm		16.67		capacitor	Solution blend mix	[116]
PVA	Graphite 1%	20	1.68 nm		6666			Solution blend mix	[81]
PVA	Expanded graphite 9%	85	33.8 nm	77.51	230	8.45	EMI-S	Solution blend mix	[117]
PVDF	Graphite 10%	10–20		35			EMI-S	Solution blend mix	[118]
Epoxy	Graphite 7%	30		21			EMI-S	Solution blend mix	[119]
PE	Colloidal graphite 34%	4	0.5–1 µm			1.95		Melt processing	[27]
PA6 PC SAM iPP	TrGO 7.5%				2.2 × 10^−8^ 4.5 × 10^−3^ 7.6 × 10^−3^ 2.5 × 10^−2^			Melt processing	[123]
PC	GNP 10%	2 to 305			1	0.71		Melt processing	[124]
PVDF	Expanded graphite 4%		<25 µm		3 × 10^−11^	0.395		Melt processing	[91]
PC	Graphite flakes 30%	44		35	0.3 × 10^−2^		EMI-S	Melt processing	[87]
PLA	Graphite 25%	6 to 150						Melt processing	[82]
LDPE	Intercalated graphite 5%					0.715		Melt processing	[92]
Epoxy	Graphite 30%	10,5						Melt processing	[26]
PA66	Graphite flakes 35%	250	Multi- layer					Melt processing	[88]
PP	Graphite 10%	300				0.63		Melt processing	[29]
NR	Expanded graphite 30%					2.371		Melt processing	[89]
EVA	GNP 30%	2	2 nm	67.6	455.85		EMI-S	Melt processing	[90]
LLDPE	Expanded graphite 25%	150		52.4	4000	19.6	EMI-S	Melt processing	[86]
TPU	Graphite flakes 30%	23		69		20.54	EMI-S	Melt processing	[125]
Asfalt	Graphite 25%	78	20–30 nm			1.95		Melt processing	[83]
PVDF TPU	Expanded Graphite 6%	297	4–7 layers	15.8 and 21.4	0.41 and 1.01		EMI-S	Ball milling	[28]
PE	Graphite 0.3%	340 nm	190 nm					Ball milling	[130]
PP	Graphite 15%	7	Few layers		4.6 × 10^−2^	6.15		Ball milling	[84]
PA6	Graphite 3%	7	Few layers	41.8	29.6	2.14	EMI-S	Ball milling	[85]
